# UV-Visible Photodetector Based on I-type Heterostructure of ZnO-QDs/Monolayer MoS_2_

**DOI:** 10.1186/s11671-019-3183-8

**Published:** 2019-12-04

**Authors:** Yong Heng Zhou, Zhi Bin Zhang, Ping Xu, Han Zhang, Bing Wang

**Affiliations:** College of Physical and Optoelectronic Engineering; College of Electronics and Information Engineering; Institute of Micro-nano Optoelectronic Technology; SZU-NUS Collaborative Innovation Centre for Optoelectronic Science & Technology; Key Laboratory of Optoelectronic Devices and Systems of Ministry of Education and Guangdong Province, Shenzhen University, Shenzhen, 518060 Guangdong People’s Republic of China

**Keywords:** Photodetector, 2D materials, I-type heterostructure

## Abstract

Monolayer MoS_2_ has shown excellent photoresponse properties, but its promising applications in high-sensitivity photodetection suffer from the atomic-thickness-limited adsorption and band gap-limited spectral selectivity. Here we have carried out investigations on MoS_2_ monolayer-based photodetectors with and without decoration of ZnO quantum dots (ZnO-QDs) for comparison. Compared with monolayer MoS_2_ photodetectors, the monolayer ZnO-QDs/MoS_2_ hybrid device exhibits faster response speed (1.5 s and 1.1 s, respectively), extended broadband photoresponse range (deep UV-visible), and enhanced photoresponse in visible spectrum, such as higher responsivity over 0.084 A/W and larger detectivity of 1.05 × 10^11^ Jones, which results from considerable injection of carries from ZnO-QDs to MoS_2_ due to the formation of I-type heterostructure existing in the contact interface of them.

## Highlights

1. Monolayer MoS_2_ has shown excellent photoresponse properties.
2. ZnO-QDs/MoS_2_ hybrid device exhibits faster response speed, extended broadband photoresponse range and enhanced photoresponse in visible spectrum.
3. I-type heterostructure existing in the contact interface of ZnO-QDs/MoS_2_.

## Introduction

Broadband photodetectors are important components of optoelectronic systems, optical communication, environmental monitoring, and so on [1–5]. Especially, UV-visible photodetectors, one of the important broadband photodetectors, have been used in biomedical imaging systems, ultraviolet astronomy, wide spectral switches, memory storage, etc. [6–8]. Therefore, it is very necessary to fabricate various materials with highly effective photoresponse in this broadband region [9, 10]. As one of the most studied transition metal dichalcogenide (TMD), 2D molybdenum disulfide (MoS_2_) has presented outstanding potential for constructing various of electronic and optoelectronic devices due to reduced dimensionality [11–13], high carrier mobility, strong electron-hole confinement, and high-light sensitivity [14–16]. However, owing to narrower band gap of 1.8 eV for the monolayer, MoS_2_ usually exhibits excellent light absorption to green light region rather than UV-visible range. In order to achieve this broadband photoresponse range, one of the most effective solutions is the construction of heterojunction with other semiconductors owning larger band gap, which can not only extend the response range to UV region from visible range but also inject photoexcited carriers so as to greatly improve the photo gain.

Type II heterojunction is the most widely studied type of two-dimensional material-based photodetectors, in which built-in electric fields can separate carriers efficiently and thus enhance photocurrent, but the recombination time of carriers is also prolonged, leading to slow response time. By comparison, the energy band structure of type I heterojunction allows charges inject from one larger band gap material to another narrower band gap material which leads to the accumulation of charges in the narrower band gap material. Moreover, charges confined inside the material can increase the carrier recombination efficiency so that devices based on it will have a faster response time. Due to the above merits, considerable attention has been paid to type I heterojunction, especially heterojunction formed between the QDs and the layered materials. These 2D-0D hybrid architectures have recently been brought into focus for their high performance as photodetectors because of this structure boosting enhancement of light absorption, facilitating band gap tunability, decreasing the response and decay time, and promoting the concentration of photoexcited charges induced by I-type heterojunction formed between the QDs and the layered materials [17–19].

Among several wide band gap semiconductors, zinc oxide (ZnO) has been a well-established material for UV photodetection due to its wide band gap (3.37 eV), high exciton binding energy (60 meV), and fast switching time on illumination with UV light [1, 20]. Recently, ZnO-QDs have widely applied in optoelectronics owing to unique optical properties, large surface-to-volume ratio, and tunable optical band gap [21, 22]. Furthermore, quantum tunneling combining with charge trapping states occurs on the surface of the ZnO-QDs in virtue of the charge carriers being confined in all the three directions. Hence, it is very crucial to present I-type hybrid heterostructure based on 2D MoS_2_ and ZnO-QDs so as to realize the excellent UV-visible broadband photoresponse with high photo absorbance, responsivity, detectivity, EQE, current on/off ratio, and so on.

Herein, we report a photodetector based on monolayer ZnO-QDs/MoS_2_ hybrid structure fabricated in a simple process. Due to the I-type heterojunction formed between monolayer MoS_2_ and ZnO-QDs, the device exhibits fast response speed, extended broadband photoresponse range (deep UV-visible), enhanced photo absorbance, photoresponse, and detectivity. It is also notable that the responsivity reaches as high as 0.084 A/W under 405-nm light at power density (PD) of 0.073 mW/cm^2^, which is comparable with that of hybrid photodetection at the same wavelength [23, 24]. Thus, our study may provide a method to improve the performance of photodetectors and expand the building blocks for high-performance optoelectronic devices.

## Method Section

### Growth of Triangular Monolayer MoS_2_

Molybdenum trioxide (MoO_3_, 99.99%) and sulfur (S, 99.5%) were used to synthesize high crystalline triangular MoS_2_ flakes on sapphires through chemical vapor deposition (CVD) procedure [7]. As substrates, sapphires were well cleaned in acetone, alcohol, and deionized water with sonication for 10 min, respectively. And then, they were tightly aligned and placed above an alumina boat which contained 3 mg MoO_3_ powders, and the boat was placed into the quartz tube and located in the high-temperature region of the furnace. Subsequently, another boat containing 120 mg sulfur (S) powders was placed into the quartz tube too and located in the lower temperature region of the furnace. Before growth, the tube was evacuated by vacuum bump and purged with pure argon (Ar) gas (99.999%) for several times in order to remove oxygen and water in the tube. Next, the temperature of the MoO_3_ powder rose to 400 °C and kept this temperature for 10 min, and then, rising to 780 °C. When it reached 650 °C, the temperature of the S powders rose to 150 °C within 5 min. Then, high- and low-temperature region remained ultimate temperatures for 5 and 15 min, respectively, and the tube was flushed with argon gas with a flow rate of 10 sccm. After the furnace was cooled down to room temperature, we got the samples grown on the substrates.

### Synthesis of ZnO Quantum Dots

ZnO quantum dots were synthesized by the sol-gel method under room temperature. A total of 0.878 g of Zinc acetate dihydrate (Zn(Ac)_2_·2H_2_O) was added in 80 ml triethylene glycol (TEG) in a conical bottle and stirred vigorously; 0.252 g of lithium hydroxide monohydrate (LiOH·H_2_O) was then gradually added into solution. After being stirred for more than 5 h, the solution became clear and green fluorescence could be observed under illumination of UV excitation. If the solution was stirred for 24 h, it exhibited much stronger fluorescence. Next, the bottle was sealed and sonicated in ice water for 30 min. And then, ethyl acetate was added into bottle until precipitates appeared. Ultimately, ZnO quantum dots powdered samples were collected by centrifugation of precipitates, washed by acetone for three times to remove unreacted precursors, heated at 70 °C for 6 h, and dispersed in ethanol for 1 h.

### ZnO-QDs/Monolayer MoS_2_ Device Fabrication

Conventional photolithography was used to directly fabricate Au/Ti electrodes on the monolayer MoS_2_ grown on the sapphire substrates to construct the devices. Positive photoresist was spin coated on sapphire at 4000 rpm for 1 min and baked at 90 °C for 1 min. And then, electrode patterns were made on monolayer MoS_2_ by photolithography system. Next, Ti film (5 nm) and Au film (50 nm) were deposited on the substrate one after another by thermal evaporation and followed by lifting off in acetone to remove Ti and Au film which adhered to photoresist so that electrodes formed. After that, devices were annealed at 200 °C for 2 h with a flow of Ar (100 sccm) to remove residues so as to form better contact between MoS_2_ and electrodes. Ultimately, the ZnO-QDs were dispersed in ethanol solution (2 mg/ml) and single droplet was dropped and spin coated over the MoS_2_ device at 1000 rpm for 60 s before baked at 70 °C for 10 min; this process being repeated 3 times to ensure that MoS_2_ surface was covered by enough ZnO-QDs.

### Characterization

Optical images were taken by Motic BA310Met to verify morphology of the as-grown MoS_2_. Atomic force microscope (AFM) height data was recorded by Bruker Dimension FastScan. Raman mapping, spectra of Raman, and photoluminescence (PL) were recorded on a Raman system (InVia-Reflex) with a 532-nm excitation laser under ambient conditions. X-ray diffraction (XRD) for crystal structure of ZnO powder samples was measured at speed of 8° min^−1^ by using D8 Advance in situ X-ray powder diffractometer. Transmission electron microscope (TEM) and high-resolution transmission electron microscopy (HRTEM) were examined by FEI Tecnai G2 F30 instrument (200 kV). The sample solutions (2 mg/ml) were dropped on carbon-coated copper grids and put into a vacuum drying oven to dry at 70 °C overnight. UV-Vis diffuse reflectance and absorption spectra were obtained by spectrophotometer (Lambda950, PerkinElmer).

### Photoelectric Performance Characterization

Our device was tested in a sealed box to prevent electromagnetic disturbance. DUV to visible lights were generated by lasers (VIASHO). Light spot with diameter of 0.7 cm was perpendicularly irradiated on device to ensure device was fully irradiated. Light power intensity was measured by a power energy meter (Thorlabs PM100D) with a silicon power head (Thorlabs S120VC). All photoelectric measurements were carried out by a source meter (Keithley 2636B).

## Results and Discussion

### Morphology and Structure of the ZnO-QDs/Monolayer MoS_2_ Photodetector

**Fig. 1:**
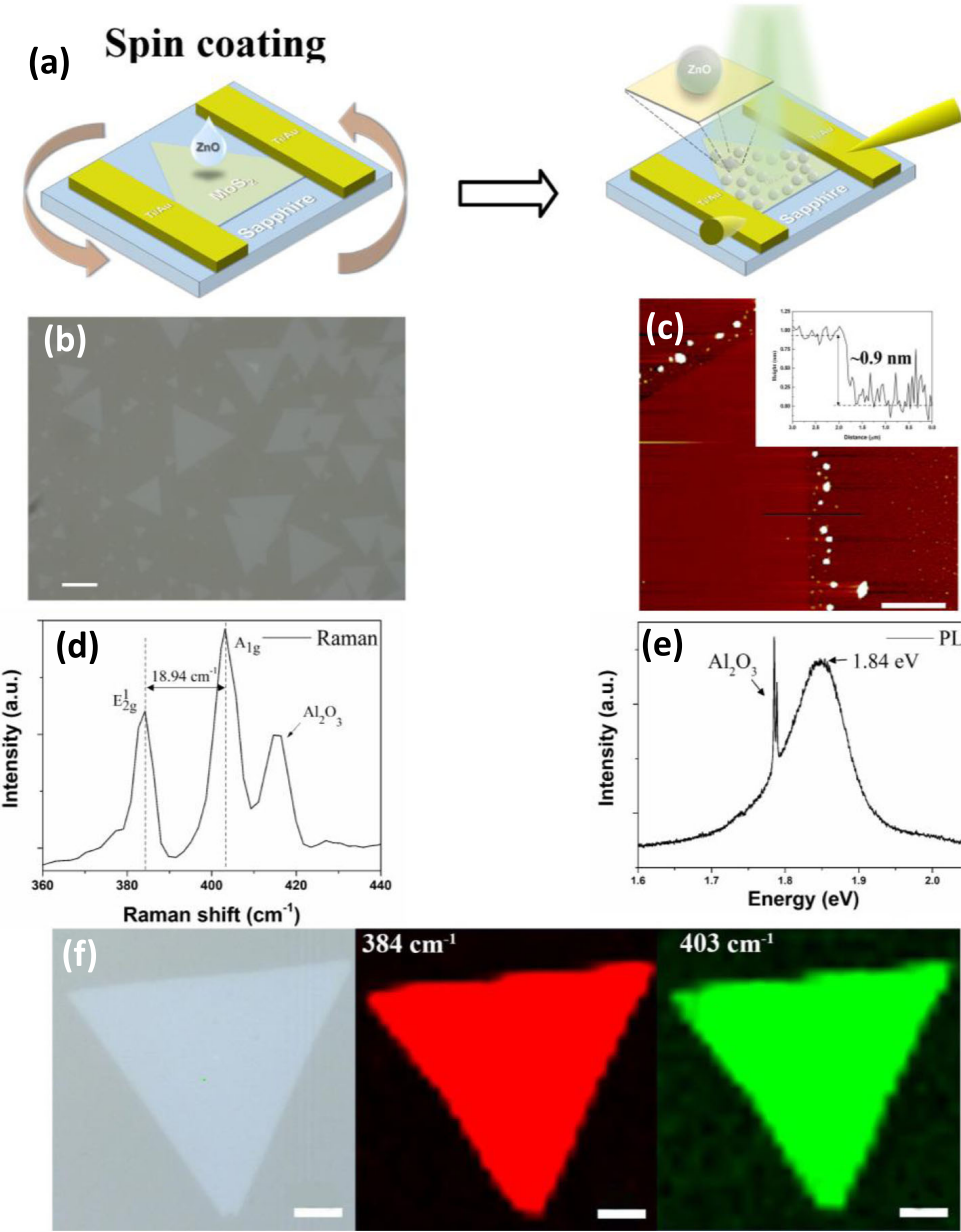
**a** Schematic diagram of pristine device and ZnO-QDs/MoS_2_ device. **b** Optical images of MoS_2_ flakes, scale bar, 10 μm. **c** AFM image, the inset shows the thickness of MoS_2_, scale bar, 2 μm. **d** Raman spectrum and **e** PL spectrum of the MoS_2_ flake. **f** Raman mapping of the MoS_2_ flake, scale bar, 5 μm

Spin coating of ZnO-QDs solution was adopted to fabricate ZnO-QDs/MoS_2_ device, as shown in Fig. 1a. Figure 1b shows the optical image of monolayer MoS_2_ flakes with average side length of 25 μm. AFM image in Fig. 1c shows the thickness of MoS_2_ flakes is ~ 0.8 nm, indicating these triangle shape MoS_2_ flakes are monolayer [25]. Additionally, two Raman active modes located at 384.24 cm^−1^ and 403.18 cm^−1^ shown in Raman spectra in Fig. 1d correspond to in-plane E^1^_2g_ and out-of-plane A_1g_, respectively. The difference of two peaks is 18.94 cm^−1^ next, in PL spectrum shown in Fig. 1e, there is a peak located at 1.84 eV. Both results are distinguishing features of monolayer MoS_2_ [26]. Corresponding Raman mapping shown in Fig. 1f indicating that the MoS_2_ flake has uniform thickness.

**Fig. 2:**
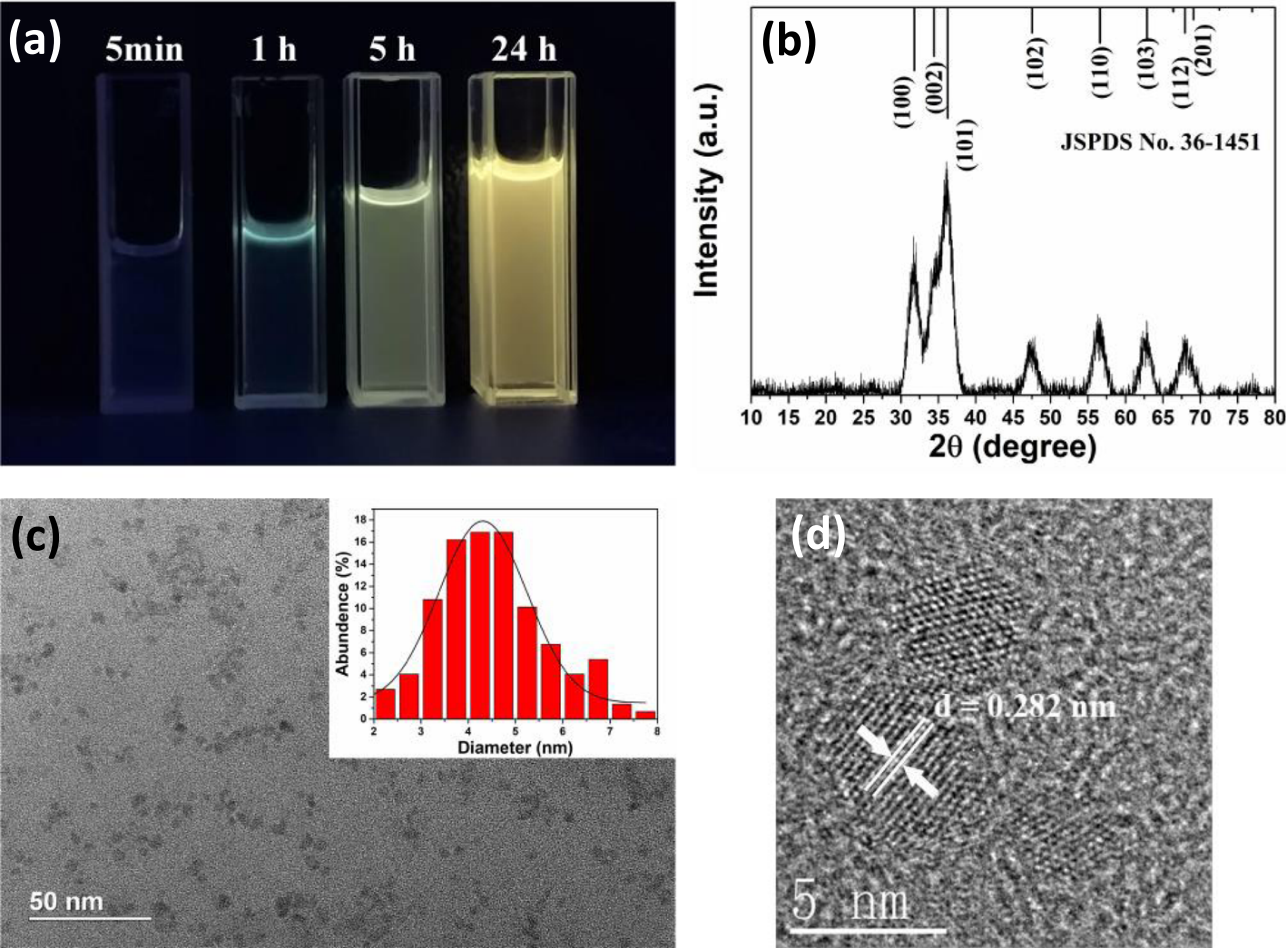
**a** ZnO-QDs with different synthesis time emitted light under illumination of UV light. **b** XRD diffraction patterns of powdered ZnO-QDs. **c**, **d** TEM and HRTEM images of ZnO-QDs. Inset shows size distribution of ZnO-QDs

ZnO-QDs can emit light due to spontaneous emission effect, which was observed in our experiment, as shown in Fig. 2b. Figure 2b demonstrates the XRD patterns of powdered ZnO-QDs that in accordance with JCPDS card no. 36-1451 and no other peaks are observed, which not only verifies the existence of ZnO but also means precursors, have been completely removed by acetone. XRD patterns of QDs materials tend to have wider full width at half maximum (FWHM) compared with bulk or powder materials [27], which is also observed in our as-fabricated ZnO powders. In order to ensure the average size and distribution of ZnO-QDs dispersed in ethanol, TEM and HRTEM are used; corresponding images of ZnO-QDs are shown in Fig. 2c and d. The average size of ZnO-QDs is 4.3 ± 1.87 nm; this result is obtained by statistical TEM analysis of over 100 ZnO-QDs. From HRTEM image in Fig. 2d, we find that ZnO-QDs have highly crystal quality with a lattice spacing of 0.28 nm, which corresponds to (100) plane of crystalline ZnO.

### Optoelectronic Performance of the ZnO-QDs/Monolayer MoS_2_ Photodetectors

**Fig. 3:**
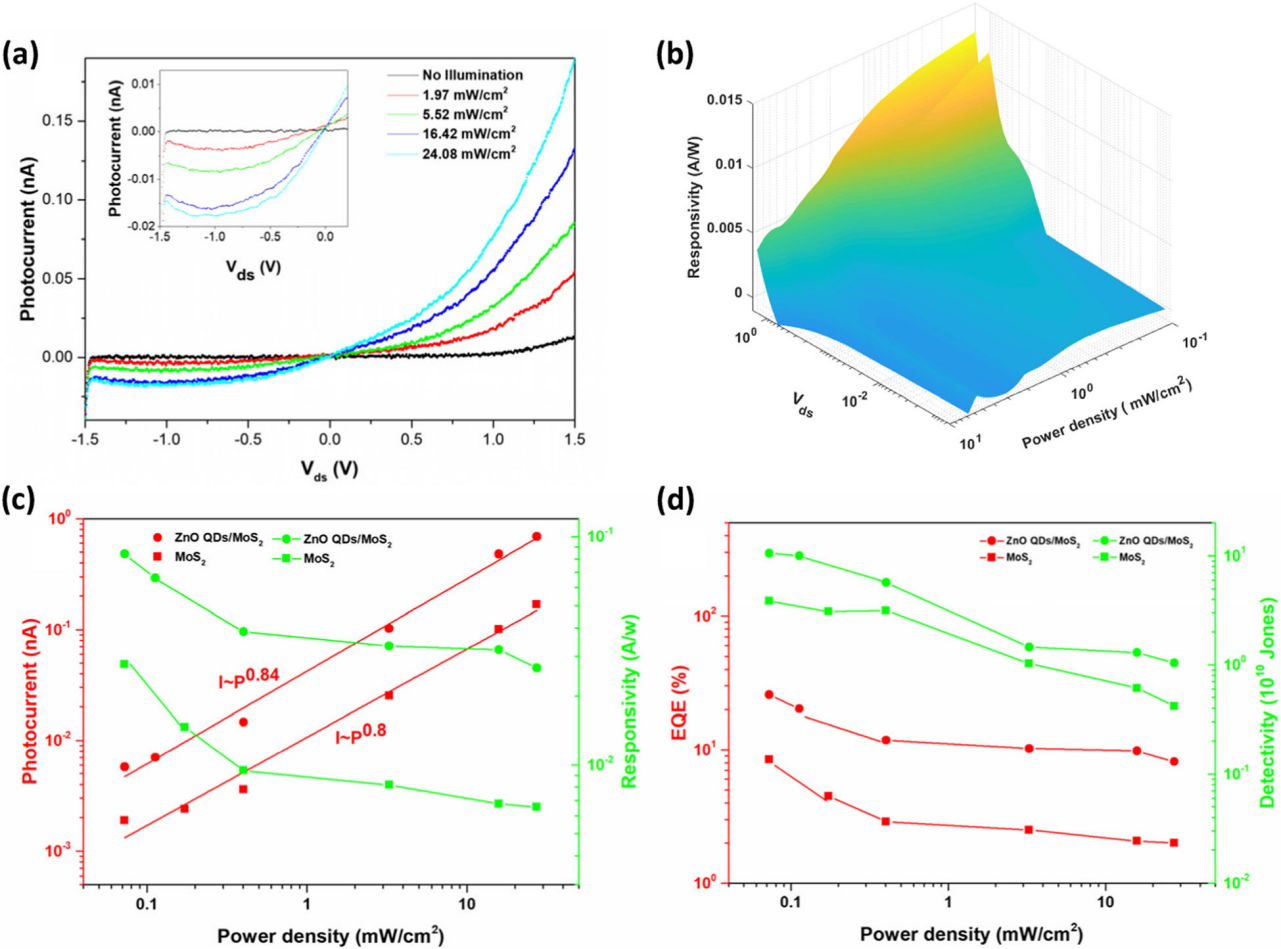
**a** I–V characteristics of the ZnO-QDs/MoS_2_ photodetector under different illumination power densities. Inset is the higher magnification I–V characteristics (negative voltage) under dark and different illumination power densities. **b** 3D responsivity map of the ZnO-QDs/MoS_2_ photodetector. **c** Power intensity-dependent photocurrent (red) and responsivity (R, green) under an excitation wavelength of 532 nm at *V*_ds_ = 1 V. **d** Power intensity-dependent external quantum efficiency (EQE, red) and specific detectivity (D*, green) at *V*_ds_ = 1 V

Current vs bias voltage (I–V) characteristics of ZnO-QDs/MoS_2_ devices in dark and under light illumination (532 nm) are shown in Fig. 3a, and the inset is higher magnification I–V characteristics located at negative voltage. The highest on/off ratio was measured to be about 100 at voltage of 0.5 V. The effective area of the device is 185.71 μm^2^ and the laser PD varies from 1.97 to 24.08 mW/cm^2^. Due to Schottky contacts between the monolayer MoS_2_ and electrodes, the I–V curves is asymmetrical. The advantages of Schottky barrier located at the contact areas are that the Schottky barrier can not only separate photogenerated electron-hole pairs in a shorter time but also reduce the electron-hole recombination rate so as to be beneficial to achieving high photocurrent and fast response speed [28–30]. As PD increased, photocurrent increased significantly when the device was applied with positive voltage, thus all further measurements were performed at *V*_ds_ = 1 V.

Responsivity is one of the crucial parameters of photodetectors, which is defined by *R*_λ_ = *I*_ph_/PS, where P is the light PD, and S is the effective area of the photosensitive. We present a 3D responsivity map of ZnO-QDs/MoS_2_ device in Fig. 3b, which reflects the impact of different *V*_ds_ and PD on responsivity. To find out the performance differences between pristine device and ZnO-QDs decorated device, we measured and compared photocurrent (*I*_ph_ = *I*_light_ − *I*_dark_) plotted by red points and responsivity (*R*_λ_) plotted by green points, under illumination of 405-nm laser with *V*_ds_ = 1 V, as shown in Fig. 3c. The photocurrent is fitted by *I*_ph_ ∼ P^α^, where P is the light PD and α represents the index of the power law. Fitting the measured photocurrents, the value of *α* = 0.8 for the pristine MoS_2_ and *α* = 0.84 for ZnO-QDs/MoS_2_ are achieved. Here, the calculated *α* close to 1 implies there are less photoexcited carrier lost due to recombination [31]. The pristine device has maximum photocurrent of 0.168 nA under laser PD of 24.08 mW/cm^2^ and exhibits responsivity of 0.028 A/W under lower laser PD of 0.073 mW/cm^2^. With the same PD, ZnO-QDs/MoS_2_ device shows a higher photocurrent of 0.667 nA and a responsivity of 0.084 A/W. This result reveals photocurrent of monolayer MoS_2_ devices can be significantly improved by decoration of ZnO-QDs. Besides, two important parameters in photodetection, external quantum efficiency (EQE) and detectivity (D*), were also calculated for further comparison. EQE is ratio of photogenerated electrons which are collected outside of the device to the number of incident photons, expressed as EQE = hc*R*_λ_/*λ*e, where h is Planck’s constant, c is the speed of light, *λ* is the wavelength of excitation light, and e is the elementary electronic charge. As for D*, it can quantify the sensitivity of the photodetector and is defined as D* = *R*_λ_S^1/2^ /(2e*I*_dark_ )^1/2^ if we assume the *I*_dark_ contributes to major noise. As shown in Fig. 3d, pristine device exhibits a maximum EQE (red) and D* (green), corresponding to 8.5% and 3.84 × 10^10^ Jones, respectively, under laser PD of 0.075 mW/cm^2^. Meanwhile, with the same PD, maximum EQE and D*, corresponding to 25.7% and 1.05 × 10^11^ Jones, respectively, both of them being about 3 times higher than those of the pristine one are obtained by ZnO-QDs/MoS_2_ device. The D* achieved by our hybrid device is competitive with that of many other reported photodetectors based on layered materials, such as graphene quantum dot/WSe_2_/Si heterojunction (4.51 × 10^9^ Jones) and graphene/graphene QDs/graphene structure (~ 10^11^ Jones) [32, 33]. This is because *I*_dark_ obtained in device is reduced to extremely small value below 0.1 nA at 1 V bias; it is comparable with *I*_dark_ of graphene-silicon heterojunction photodetector (0.1 nA at zero bias) [34].

**Fig. 4:**
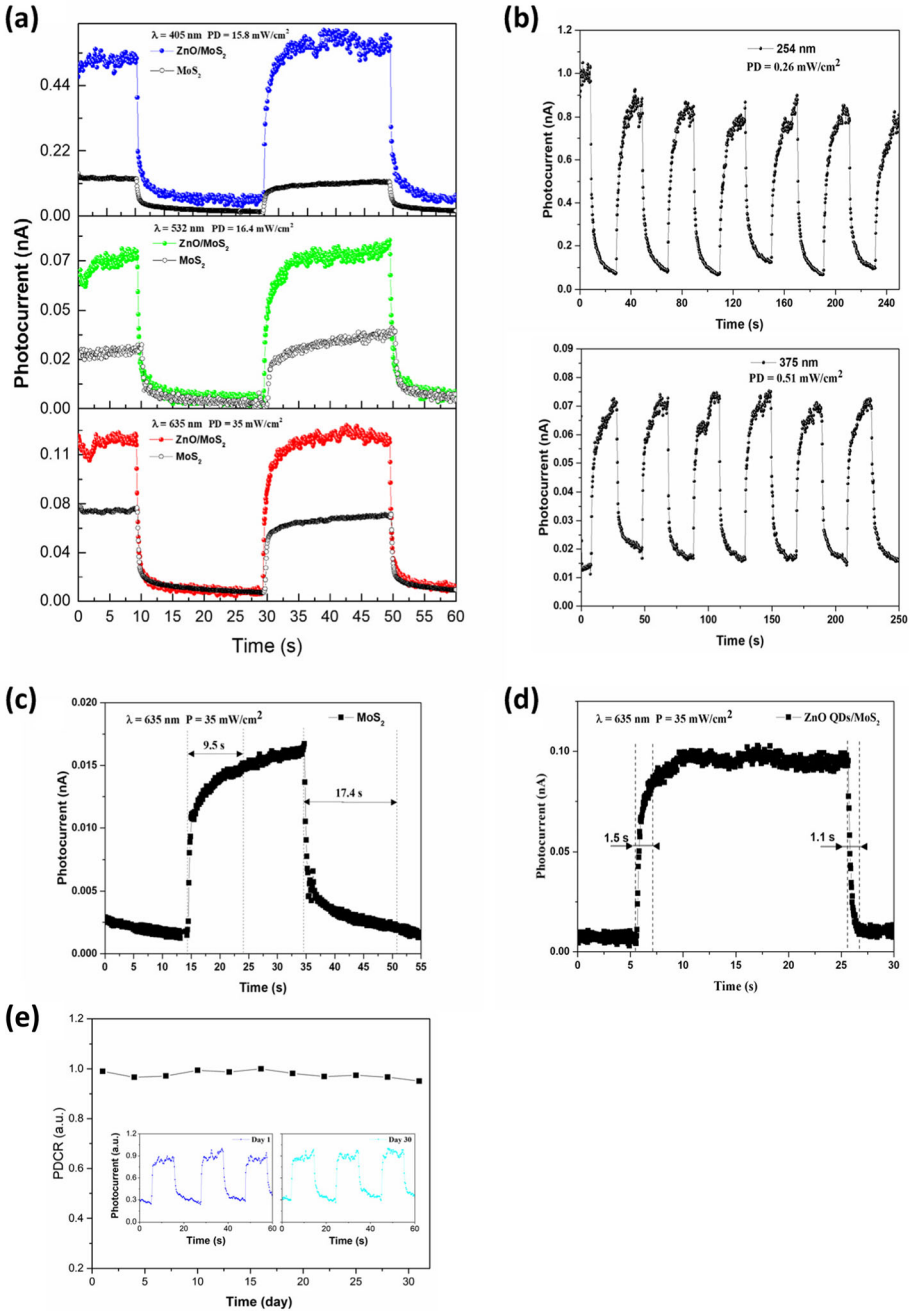
**a** Photocurrent of pristine device and ZnO-QDs/MoS_2_ photodetector illuminated under different PD with different wavelength at *V*_ds_ = 1 V. **b** Multiple cycles of photoresponse of the ZnO-QDs/MoS_2_ photodetector under DUV (254 nm) and UV (375 nm) light illumination (*V*_ds_ = 1 V). The response time of **c** pristine device and **d** ZnO-QDs/MoS_2_ photodetector illuminated under PD of 35 mW/cm^2^ with wavelength of 635 nm at *V*_ds_ = 1 V. **e** Normalized PDCR of ZnO-QDs/MoS_2_ photodetector under a 532-nm laser illumination at *V*_ds_ = 1 V; photocurrent measured at day 1 and day 31 was inserted for better comparison

Photocurrent of pristine device and ZnO-QDs decorated device under exposure of laser with different wavelength are given in Fig. 4a. There exists clear enhancement of photocurrent whatever in 405 nm, 532 nm, or 635 nm, which implies ZnO quantum dots with a wide band gap are able to improve the performance of visible light detection. We further investigated the broadband spectral response of the hybrid device, 254-nm light with PD of 0.26 mW/cm^2^ and 375-nm light with PD of 0.51 mW/cm^2^ were applied to illuminate hybrid device and excellent photoresponse properties were observed as shown in Fig. 4b. Besides, the hybrid device shows no response when it was illuminated by the light with wavelength over 800 nm. Although the power of UV light illumination is low, the photocurrent is still much higher or at least comparable with those obtained under visible light illumination with much higher PD. We believe it is the wide band gap of ZnO-QDs that allows hybrid device to absorb more photons when UV light falls on it; thus, lots of carries are generated and transferred to MoS_2_ so as to greatly enlarge photocurrent. Moreover, after switching on/off status for 6 times over 250 s, photocurrent and dark current still stayed on their level, which demonstrated excellent photo stability of this hybrid device.

A photodetector with fast response speed is suitable of some areas, such as optical communication and video imaging. As another important parameter of photodetectors, response time was also investigated under incident 635-nm light with PD of 35 mW/cm^2^. In this work, we defined the rise time and the decay time of the photodetector as the time taken for the device to reach 90% of the equilibrium value from the initial current, and vice versa, respectively. For the pristine device, rise time was 9.5 s and decay time was 17.4 s, such slow response speed mainly due to trap states located in band gap which was introduced by defects in the materials [35]. After MoS_2_ photodetector was decorated with ZnO-QDs, as shown in Fig. 4c and d, the rise and decay time reduced to 1.5 s and 1.1 s, the response time was reduced by 84.2% and 93.7%, respectively. This result demonstrates that ZnO-QDs can greatly shorten response time of MoS_2_ photodetectors and make this hybrid photodetector a suitable candidate for practical applications. To evaluate long-term stability of the hybrid photodetector, we measured the photocurrent of the device over 1 month (interval of 3 days), as shown in Fig. 4e; photocurrent/dark current ratio (PDCR = *I*_ph_/*I*_dark_) was applied. After being exposed to air for 1 month, PDCR of the device shows no obvious degeneration; inserted images show that the current measured at day 1 and day 31 almost remains on the same level; clearly, this hybrid photodetector has good stability for long-term photodetection.

### Photoresponse Mechanism

**Fig. 5:**
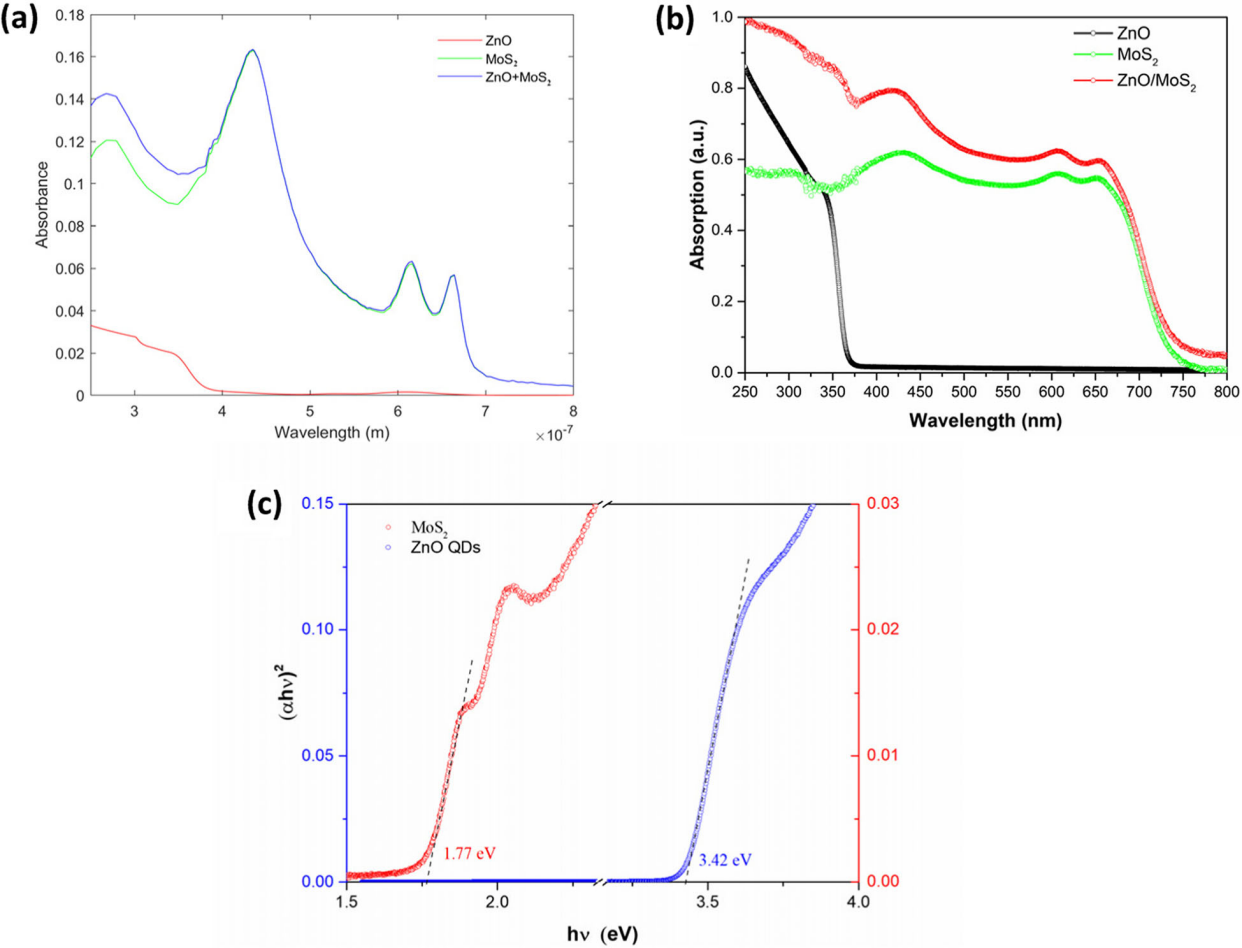
**a** Calculated absorption spectra of MoS_2_, ZnO-QDs, and ZnO-QDs/MoS_2_. **b** UV-vis absorption spectra of MoS_2_, ZnO-QDs, and ZnO-QDs/MoS_2_. **c** Tauc plots of MoS_2_ and ZnO-QDs

Here we investigated the mechanism on the photodetection performance enhancement of the ZnO-QDs/MoS_2_ photodetector. Firstly, we checked the absorption of the hybrid structure by means of numerical simulations. Using the finite element method, we built a calculation model that consists of an air domain on top and a sapphire substrate at beneath. The top and bottom of the model were truncated by two perfectly matched layers to avoid spurious reflections. The refractive index of the sapphire substrate was set as a constant 1.75. In the beginning, we respectively put 0.8-nm-thick MoS_2_ layer and 4.5-nm-thick ZnO layer on the sapphire substrate, to check the independent absorptions. The refractive index of MoS_2_ monolayer was taken from reference [36], and the one for ZnO was taken from reference [37]. We then put a hybrid layer (ZnO over MoS_2_) on the same sapphire substrate to examine the overall absorption. As shown in Fig. 5a, the hybrid layer presents an enhanced absorption at the wavelength region below than 400 nm compared with the bare MoS_2_ monolayer, revealing a better UV absorption after the ZnO-QDs decoration. Then, we experimentally checked UV-Vis absorption spectra of MoS_2_, ZnO-QDs, and ZnO-QDs/MoS_2_ and the result is matched to the calculated one. As shown in Fig. 5b, monolayer MoS_2_ exhibits a broadband absorption range from UV to visible wavelength light and no absorption peaks are found when the wavelength increases to NIR. As for ZnO-QDs, the absorption peaks are located at UV wavelength light and there exists a larger absorption rate in comparison with MoS_2_. After decoration of ZnO-QDs, we found that ZnO-QDs/MoS_2_ exhibits stronger absorption ability than pristine MoS_2_, indicating that the heterostructure has more intensive light-matter interaction. The corresponding Tauc plots are shown in Fig. 5c, and the band gap of MoS_2_ and ZnO-QDs can be calculated to be ~ 1.77 eV and ~ 3.42 eV, respectively, which are close to the values in the previous reports [27, 38].

**Fig. 6:**
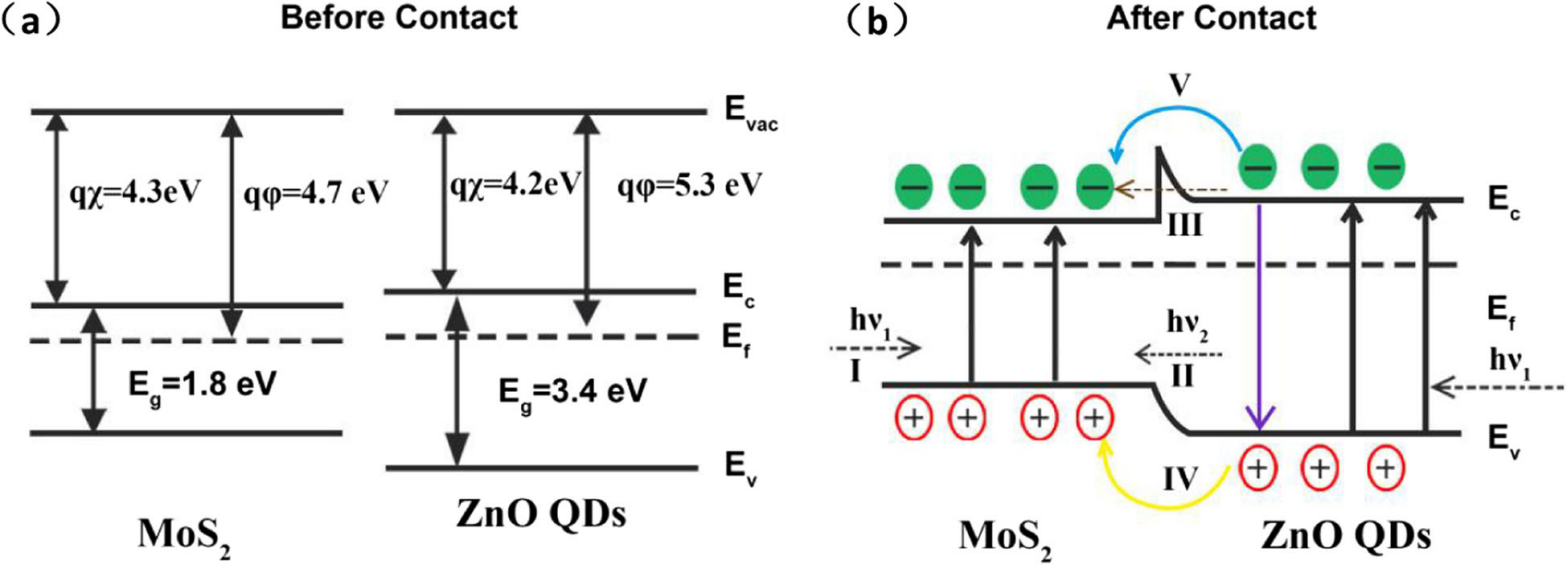
**a** and **b** are band diagrams of MoS_2_ and ZnO-QDs before and after contact

Based on the previous reports, MoS_2_ and ZnO-QDs are both n-type semiconductors with the work function of 4.7 eV and 5.3 eV [39, 40], respectively. Electron affinity of MoS_2_ is approximately 4.3 eV [41], which is slightly larger than that of ZnO-QDs (4.2 eV) [42]. Band gap of MoS_2_ and ZnO-QDs is considered to be 1.8 eV and 3.4 eV according to Tauc plot calculations. The energy band structure of MoS_2_ and ZnO (before and after contact), shown in Fig. 6a and b, is constructed and used to investigate the mechanism about the photodetection performance enhancement.

MoS_2_/ZnO-QDs heterojunction is formed by Van der Waals forces and the I-type band alignment at the interface can be used to explain the enhanced responsivity [42]. When these heterostructures are illuminated under UV light, both ZnO-QDs and MoS_2_ strongly absorb light photon and electrons move from valence to conduction band so that the process “I” occurs. Afterwards, electrons are injected from ZnO-QDs conduction band into MoS_2_ conduction band so as to form process “V” through thermal agitation, meanwhile, part of electrons in ZnO-QDs conduction band tunnel to conduction band in MoS_2_, resulting in the process “III.” And then, holes in ZnO-QDs valence band move to the corresponding valence band in MoS_2_, as shown in the process “IV.” Also, spontaneous emission can make some of the electrons in the conduction band of ZnO-QDs and move back to the valence band to emit photon that can excite electrons in the valence band of MoS_2_ to the conduction band so as to form the process “II.” On the other hand, similar processes happened when the hybrid devices were illuminated by visible light, except the excited electrons were from defect energy level of ZnO-QDs [43], which would lower excited energy. As a result, these excited electron-hole pairs transfer from ZnO-QDs to MoS_2_ and lead to significant enhancement of photocurrent in comparison with the pristine device. In addition, large number of excited carries MoS_2_ will greatly increase the recombination rate and decrease the response and decay time [42], which were observed in Fig. 4c and d.

## Conclusions

In summary, we report a photodetector based on monolayer MoS_2_/ZnO-QDs hybrid structure. Compared with the monolayer MoS_2_, ZnO-QDs decoration leads to not only huge enhancement of photoresponse in visible spectrum but also extension to deep ultraviolet (DUV) range. Under excitation of visible light, this hybrid device exhibits faster response speed (1.5 s and 1.1 s, respectively), higher responsivity over 0.084 A/W, and larger detectivity of 1.05 × 10^11^ Jones. These are attributed to large number of injection of carries from ZnO-QDs to MoS_2_. In addition, the hybrid device shows excellent stability under exposure to atmosphere at room temperature. Thus, our study may provide a method to improve the performance of photodetectors and expand the building blocks for high-performance optoelectronic devices.

## Data Availability

In the manuscript, all data supporting their findings are from the fabrication experimental, characterization, and measurement. All authors wish to share their data. The data can be shared.

## Abbreviations

PL: Photoluminescence
AFM: Atomic force microscopy
XPS: X-ray photoelectron spectra
CVD: Chemical vapor deposition
*R*_λ_: Responsivity
*I*_ds_: Source-drain current
*I*_ph_: Photocurrent
EQE: External quantum efficiency
D*: Detectivity
VB: Valence band
CB: Conduction band
